# Preliminary list of horse flies (Diptera, Tabanidae) of Serbia

**DOI:** 10.3897/zookeys.117.1328

**Published:** 2011-07-08

**Authors:** Stjepan Krčmar

**Affiliations:** Department of Biology, J.J. Strossmayer University of Osijek, Trg Lj. Gaja 6, HR-31000 Osijek, Croatia

**Keywords:** Tabanidae, Diptera, Serbia, Europe

## Abstract

Thirty six species of horse flies (Tabanidae) were previously known from Serbia (Europe). The present faunistic study of horse flies (Tabanidae) has resulted in the recording of the 4 new species *Atylotus fulvus* (Meigen, 1804); *Tabanus miki* Brauer in Brauer and Bergenstamm, 1880; *Tabanus unifasciatus* Loew, 1858; and *Heptatoma pellucens* (Fabricius, 1776), in the fauna of Serbia. The genus *Heptatoma* Meigen, 1803 is cited for the first time in the fauna of Serbia. 40 species are currently known from Serbia, belonging to nine genera. The fauna can be considered relatively poorly studied. Most of the species belong to the Boreal-Eurasian type of fauna 23, followed by the South European group with 8 species, the Mediterranean group with 6 species, European group with 2 species and Central European group with 1 species.

## Introduction

The Tabanidae family contains over 4,000 described species found throughout the world (Chainey 1993). The females are known as mechanical vectors of viruses, bacteria, protozoans and helminths that cause various diseases in wild and domestic animals (Foil 1989, Desquesnes and Dia 2004). Therefore, during the last thirty years numerous studies into the effectiveness of synthetic and natural attractants in the sampling of horse flies have been carried out around the world (French and Kline 1989, Phelps and Holloway 1992, Hribar et al. 1992, Hayes et al. 1993, Leprince et al. 1994, Krčmar et al. 2005, 2006, Krčmar 2007, Mihok et al. 2007, Cilek and Olson 2008, Mihok and Mulye 2010). Moreover, in this period a few new traps for collecting horse flies have been made (Hribar et al. 1991, Cilek and Medrano 2000, Mihok 2002, Dia et al. 2004, Mihok et al. 2006). However, there are some regions in the world that have not yet been sufficiently studied, one of these regions is the Balkan Peninsula. The horsefly fauna is poorly known in Central Balkan countries, 40 species were recorded in Macedonia, 42 species in Montenegro and 36 species in Serbia (Strobl 1898, 1900, 1902, Doflein 1921, Leclercq 1959, 1960a, 1960b, 1965, 1966, 1968, Coe 1958, 1960, Moucha 1959, 1965, Moucha and Chvála 1964, Majer 1985, Krčmar et al. 2002, Zeegers 2005, www.faunaeur.org). At the beginning of the twentieth century there were a few sporadic visits by foreign entomologists to Central Balkan countries, during which time several species of horse flies were collected. Most horse flies in the Central Balkan countries were collected after the Second World War, during the sixties and during the study tour of Czech and Belgian entomologists in the countries of Southeastern Europe. During my visits to Serbia in 2004 and 2006, I collected a few interesting species of horse flies, which led me to summarize all available data on the horsefly fauna of Serbia. Because of this, this work is based on literature findings and data obtained from a faunal survey conducted in the summer of 2004 and during the spring and summer months of 2006.

## Material and methods

Samplings of horse flies in Serbia were carried out during 2004 and 2006 mostly in the area of the Fruška Gora national park. In this period, horse flies were collected at 8 localities. The Fruška Gora national park is an isolated, narrow, mainland mountain in the Pannonian plain. Most of the mountain lies in Vojvodina, Serbia except for a small section to the west which lies in Croatia. To the north, the mountain is bordered by the Danube. Lengthwise, it is approximately 80 km east to west and 15 km north to south (45°10'0"N, 19°40'0"E). Its highest peak is Crveni Čot at 539 m (http://en.wikipedia.org). Its location, specific geological history and different microclimatic conditions make it very interesting and important to science. Thanks to the unique and very rich deposits of fossil fauna and flora, Fruška Gora is called the mirror of the geological past. The main characteristic of this region is the existence of numerous protected, rare and endangered species (http://www.npfruskagora.co.rs). The horse flies were collected on 25 June 2004, 24 July 2004, 20 May 2006, 24 July 2006, and 10 August 2006 from horses by hand and by means of a sampling net when horse flies flew into a car. All collected horse flies were preserved in ethanol. Identification and nomenclature followed that of Chvála et al. (1972), Chvála (1988) and Mally (1987). Also, the presence of some species was determined upon a review of literature data. The full scientific names for all species including the author and year is only provided in the updated list of Serbian Tabanidae and is omitted from the text below.

## Results of the study at Fruška Gora

All together 542 specimens were collected (Table 1) belonging to 24 species of horse flies grouped into the subfamilies Chrysopsinae and Tabaninae and the genera: *Chrysops*, *Atylotus*, *Hybomitra*, *Tabanus*, *Heptatoma* and *Haematopota*. Four species: *Tabanus glaucopis*, *Tabanus exclusus*, *Haematopota pluvialis*, and *Tabanus tergestinus* made up 81% of the fauna of horse flies in the researched area, while 19% were representatives of other species (Table 1). The most numerous genus is *Tabanus* with 11 species, followed by *Haematopota* with 4 species, *Atylotus* and *Hybomitra* with 3 species, *Chrysops* with 2 species and *Heptatoma* with 1 species (Table 1). From the collected sample, 4 species of horse flies new to the fauna of Serbia were determined, these are *Atylotus fulvus*, *Tabanus miki*, *Tabanus unifasciatus*, and *Heptatoma pellucens*. Four females of the species *Atylotus fulvus* were collected in Letenka on 24 July 2006. One female of the species *Tabanus miki* was collected in Brankovac on 24 July 2006. The third new species *Tabanus unifasciatus* was collected in Brankovac on 24 July 2006 (1♀), and 10 August 2006 (3♀), while two females were collected at the locality in Zmajevac on 10 August 2006. Finally, the fourth species is *Heptatoma pellucens* that was collected at Brankovac on 10 August 2006. Thanks to the kindness of Dr. Th. Zeegers and the data he provided for this manuscript four additional horse fly species are added to the Serbian fauna: *Therioplectes tunicatus*, *Hybomitra aterrima*, *Hybomitra micans* and *Dasyrhamphis umbrinus*. Two females of *Hybomitra aterrima* were collected at the locality in Kopaonik, Jankova Bara on 11 June 2009 (Th. Zeegers unpublished data through personal communication). Also, one female specimen of *Therioplectes miki* was collected at the locality in Kopaonik, Lisina on 12 June 2009 (Th. Zeegers unpublished data through personal communication). Most of the species belong to the Boreal-Eurasian type of fauna (n= 23), (Olsufjev 1977). These species are: *Chrysops caecutiens*, *Chrysops relictus*, *Chrysops rufipes*, *Chrysops viduatus*, *Atylotus fulvus*, *Atylotus rusticus*, *Hybomitra aterrima*, *Hybomitra bimaculata*, *Hybomitra ciureai*, *Hybomitra distinguenda*, *Hybomitra muehlfeldi*, *Tabanus autumnalis*, *Tabanus bovinus*, *Tabanus bromius*, *Tabanus cordiger*, *Tabanus glaucopis*, *Tabanus maculicornis*, *Tabanus miki*, *Tabanus sudeticus*, *Heptatoma pellucens*, *Haematopota italica*, *Haematopota pluvialis*, and *Haematopota subcylindrica*. The following 6 are Mediterranean species: *Chrysops flavipes*, *Therioplectes tunicatus*, *Tabanus promesogaeus*, *Tabanus exclusus*, *Tabanus lunatus*, and *Philipomyia graeca* (Olsufjev 1977, Chvála et al. 1972). Furthermore, the following 8 are Southern European species: *Atylotus loewianus*, *Tabanus quatuornotatus*, *Tabanus tergestinus*, *Tabanus unifasciatus*, *Haematopota bigoti*, *Haematopota ocelligera*, *Haematopota pandazisi*, *Dasyrhamphis umbrinus* (Olsufjev 1977, Chvála et al. 1972). Only, *Hybomitra micans*, and *Hybomitra pilosa* belong to the group of European species (Chvála et al. 1972), while *Therioplectes gigas* belong to Central European group of species (Zeegers 2005).

The following list of species includes all available literature records and new records based on the study at Fruška Gora and previously unpublished records provided by Dr. Theo Zeegers.

**Table 1.** Species and numbers of horse flies collected in Serbia during 2004 and 2006.

Species	No. of Specimens	%
*Tabanus glaucopis*	231	42.61
*Tabanus exclusus*	98	18.08
*Haematopota pluvialis*	63	11.62
*Tabanus tergestinus*	45	8.30
*Haematopota bigoti*	26	4.79
*Tabanus bromius*	19	3.50
*Tabanus sudeticus*	14	2.58
*Hybomitra ciureai*	12	2.21
*Tabanus unifasciatus*	6	1.10
*Tabanus autumnalis*	4	0.73
*Atylotus rusticus*	4	0.73
*Atylotus fulvus*	4	0.73
*Tabanus maculicornis*	2	0.36
*Atylotus loewianus*	2	0.36
*Chrysops caecutiens*	2	0.36
*Haematopota italica*	2	0.36
*Chrysops viduatus*	1	0.18
*Hybomitra bimaculata*	1	0.18
*Hybomitra solstitialis*	1	0.18
*Tabanus bovinus*	1	0.18
*Tabanus cordiger*	1	0.18
*Tabanus miki*	1	0.18
*Heptatoma pellucens*	1	0.18
*Haematopota pandazisi*	1	0.18
Total: 24	542	

### List of Tabanidae species recorded in Serbia.

**Subfamily Chrysopsinae**

**Genus** Chrysops Meigen, 1803

1. *Chrysops caecutiens* (Linnaeus, 1758)

2. *Chrysops flavipes* Meigen, 1804

3. *Chrysops relictus* Meigen, 1820

4. *Chrysops rufipes* Meigen, 1820

5. *Chrysops viduatus* (Fabricius, 1794)

**Subfamily Tabaninae**

**Genus** AtylotusOsten – Sacken, 1876

6. *Atylotus fulvus* (Meigen, 1804)

7. *Atylotus loewianus* (Villeneuve, 1920)

8. *Atylotus rusticus* ( Linné, 1767)

**Genus** Therioplectes Zeller, 1842

9. *Therioplectes gigas* (Herbst, 1787)

10. *Therioplectes tunicatus* Szilády, 1927

**Genus** Hybomitra Enderlein, 1922

11. *Hybomitra aterrima* (Meigen, 1820)

12. *Hybomitra bimaculata* (Macquart, 1826)

13. *Hybomitra ciureai* (Séguy, 1937)

14. *Hybomitra distinguenda* (Verrall, 1909)

15. *Hybomitra micans* (Meigen, 1804)

16. *Hybomitra muehlfeldi* (Bauer *in* Brauer and Bergenstamm, 1880)

17. *Hybomitra pilosa* (Loew, 1858)

**Genus** Tabanus Linnaeus, 1758

18. *Tabanus autumnalis* Linnaeus, 1761

19. *Tabanus bovinus* Linnaeus, 1758

20. *Tabanus bromius* Linnaeus, 1758

21. *Tabanus cordiger* Meigen, 1820

22. *Tabanus exlusus* Pandellé, 1883

23. *Tabanus glaucopis* Meigen, 1820

24. *Tabanus lunatus* Fabricius, 1794

25. *Tabanus maculicornis* Zetterstedt, 1842

26. *Tabanus miki* Brauer *in* Brauer and Bergenstamm, 1880

27. *Tabanus promesogaeus* Mally, 1987

28. *Tabanus quatuornotatus* Meigen, 1820

29. *Tabanus sudeticus* Zeller, 1842

30. *Tabanus tergestinus* Egger, 1859

31. *Tabanus unifasciatus* Loew, 1858

**Genus** Heptatoma Meigen, 1803

32. *Heptatoma pellucens* (Fabricius, 1776)

**Genus** Haematopota Meigen, 1803

33. *Haematopota bigoti* Gobert, 1880

34. *Haematopota italica* Meigen, 1804

35. *Haematopota ocelligera* (Kröber, 1922)

36. *Haematopota pandazisi* (Kröber, 1936)

37. *Haematopota pluvialis* (Linnaeus, 1758)

38. *Haematopota subcylindrica* Pandellé, 1883

**Genus** Philipomyia Olsufjev, 1964

39. *Philipomyia graeca* ( Fabricius, 1794)

**Genus** Dasyrhamphis Enderlein, 1922

40. *Dasyrhamphis umbrinus* (Meigen, 1820)

## Discussion

A previous list of Tabanidae of Serbia was based on literature data from previous studies (Strobl 1900, 1902, Coe 1958, 1960, Moucha 1959, 1965, Moucha and Chvála 1964, Leclercq 1966, 1968, Krčmar et al. 2002, Zeegers 2005, www.faunaeur.org). According to these studies 36 species were mentioned from Serbia. Four species were recorded as new for the fauna of Serbia during this study; two of them belong to genus *Tabanus*, followed by the genera *Atylotus* and *Heptatoma* with one species. All new species for the fauna of Serbia were collected during field work. New species were mainly collected on the localities of the Fruška Gora national park. The analysis of the recorded species during the 2004 and 2006 study resulted in a very high percent of Mediterranean species *Tabanus exclusus* in localities of Fruška Gora. *Tabanus exclusus* was represented with 18% in the collected sample indicating different microclimatic conditions. Interesting data for comparison with this high percent of records of *Tabanus exclusus* in Fruška Gora is that this species in the Mediterranean part of Croatia was the most common with 21% (Krčmar 1999). Furthermore, very few specimens of *Tabanus exclusus* were collected on the southern slopes of the mountain massifs of Dilj, Krndija and Papuk in the continental part of Eastern Croatia (Krčmar and Mikuska 2001). The distribution of this species belong to the area of Southern Europe and South Eastern Europe (Chvála et al. 1972). All these comparisons confirmed that Fruška Gora is very interesting and important for faunistical studies. Only Strobl (1900) and Moucha (1959) mentioned the presence of species *Chrysops relictus* in Serbia but gave no other data except the name of country where the horse flies were collected, which they marked as “Serbia”. Also, there are no exact data about the dates of collection, for the species *Therioplectes gigas* and *Tabanus lunatus* (Strobl 1902, Moucha 1959). However, two years ago on 8 June 2009 one female of the species *Therioplectes gigas* was collected on Stara Planina 35 km ENE of Pirot (Th. Zeegers unpublished data through personal communication). The Tabanidae fauna of Serbia is very poor compared with neighboring countries (e.g., Croatia 78 species, Bosnia and Herzegovina 62 species). The most recent published article about Tabanidae fauna of Serbia was from the 1960s, where all the records were summarized as horse flies from Yugoslavia. The 40 determined species of horse flies indicate the necessity to continue with faunistical research, because this is certainly not the final number of horse flies in Serbia, the occurrence of many additional species is expected.
